# Validation of diagnostic accuracy using digital slides in routine histopathology

**DOI:** 10.1186/1746-1596-7-35

**Published:** 2012-03-31

**Authors:** László Fónyad, Tibor Krenács, Péter Nagy, Attila Zalatnai, Judit Csomor, Zoltán Sápi, Judit Pápay, Júlia Schönléber, Csaba Diczházi, Béla Molnár

**Affiliations:** 1 First Department of Pathology and Experimental Cancer Research, Semmelweis University, Budapest, Hungary; 2 Second Department of Internal Medicine, Semmelweis University, Budapest, Hungary

**Keywords:** Retrospective study, Surgical pathology, Diagnostic error, Optical microscopy, Whole slide imaging

## Abstract

**Background:**

Robust hardware and software tools have been developed in digital microscopy during the past years for pathologists. Reports have been advocated the reliability of digital slides in routine diagnostics. We have designed a retrospective, comparative study to evaluate the scanning properties and digital slide based diagnostic accuracy.

**Methods:**

8 pathologists reevaluated 306 randomly selected cases from our archives. The slides were scanned with a 20× Plan-Apochromat objective, using a 3-chip Hitachi camera, resulting 0.465 μm/pixel resolution. Slide management was supported with dedicated Data Base and Viewer software tools. Pathologists used their office PCs for evaluation and reached the digital slides via intranet connection. The diagnostic coherency and uncertainty related to digital slides and scanning quality were analyzed.

**Results:**

Good to excellent image quality of slides was recorded in 96%. In half of the critical 61 digital slides, poor image quality was related to section folds or floatings. In 88.2% of the studied cases the digital diagnoses were in full agreement with the consensus. Out of the overall 36 incoherent cases, 7 (2.3%) were graded relevant without any recorded uncertainty by the pathologist. Excluding the non-field specific cases from each pathologist's record this ratio was 1.76% of all cases.

**Conclusions:**

Our results revealed that: 1) digital slide based histopathological diagnoses can be highly coherent with those using optical microscopy; 2) the competency of pathologists is a factor more important than the quality of digital slide; 3) poor digital slide quality do not endanger patient safety as these errors are recognizable by the pathologist and further actions for correction could be taken.

**Virtual slides:**

The virtual slide(s) for this article can be found here: http://www.diagnosticpathology.diagnomx.eu/vs/1913324336747310.

## Introduction

Using still digital images in pathology for various purposes became increasingly popular and become essential as an easy way to archive and share medical information. The first telepathology networks to provide pathological diagnosis and consultation to remote sites used still images as well [1]. The limitations of these telepathology networks (i.e. the lack of sufficient number and quality of images) were obvious and resulted in diagnostic errors [2]. Later hybrid dynamic/store-and-forward telepathology systems were tested, where pathologists remotely controlled robotised microscopes. Results were impressive, though disadvantages of this solution, such as the dependence on the assistance in situ to handle the glass slides, interfered with the real success of this solution [3]. Digital slides (DS) that offer entire slide for dynamic access via the computer and its monitor overcome the limitations imposed by static preselected microscopical frames [4]. The initial enthusiasm for DS in daily use was soon over as serious constraints, mainly related to information technology (IT), rather than the diagnostic accuracy were revealed, such as the issue of proper and cheap storage capacity, slow processors compared to the requirements of DS and the concern of the proper resolution of monitors [5-8].

Although the above problems have been more or less solved by now, the real revolution of DS is waiting, as their use is still limited in certain fields [9]. In science where the regulations are not as strict as in health care, the success of DS is unquestionable and offers additional benefits (e.g. the core finding on a TMA digital slide is easier, while on a FISH-TMA slide is actually impossible without fluorescens-scanning of the whole slide) [10]. Another unique attribute - the possibility to standardize teaching materials - was the motive for spreading DS in education [11]. Furthermore DS are increasingly involved in quality control (QC) of the pathology work flow [12,13]. This is an obvious paradox condition that we trust the quality of DS for QC of optical slides (OS), but we are still reluctant to accept DS equivalently for the routine pathology practice.

In this paper we report a retrospective, comparative study evaluating the scanning properties and accuracy of DS-based diagnostic workflow. Reviewing the literature and based on our unpublished, pilot studies we have designed a method that could be useful for other laboratories as well to define the possible sources of diagnostic pitfalls in a digital workflow. We have estimated the major causes of dissatisfaction that could explain the lack of trust in DS, and the delay in the general breakthrough of this technique in the routine practice.

Our questions were:

1. Can DS based diagnostic work flow, supported by dedicated software tools for database integration and microscopic analysis help pathologists to overcome their reluctance against DM?

2. Is it possible to estimate the type of errors that cause the misdiagnosis?

3. Can we define a list of samples according to the origin, where DS are suitable to be used in routine practice and define those where it is not recommended?

4. Do pathologists' interpretative skills and experience affect the diagnostic results using DS and how important these factors are comparing to the actual quality of DS?

## Materials and methods

### Participants

Seven experienced pathologists and a junior pathologist participated in the study. The seniors have been working as consultants for 13-28 years (mean: 21.). The participants will be denoted as PathA-PathH. They are specialized in various fields of pathology, respectively haematopathology, liver pathology, pulmonary pathology, soft tissue pathology, breast pathology, kidney pathology, gastrointestinal and pancreas pathology. All had experience with DS as they participated in our pilot studies and except PathA and PathD have been exclusively using DS for graduate teaching, since the academic year 2007-2008. Two technicians were responsible for slide scanning, database and network management.

### Case selection and signing out

306 cases (125 biopsy specimens and 181 surgical resection specimens) from 1998 to 2007 (Table 1) were selected from our archive (routine and consultation cases) achieving notable washout period for the pathologists. For comparison our Institute had 13300 own and 1500 consultant cases in 2007. Case selection was randomised. By the SNOMED-L/M codes cases were listed from our LIS. Simple cases (e.g. appendicitis, cholecystitis) were sorted out to enrich the challenging cases in the study set. 1858 slides (1062 H&E, 90 Giemsa, 533 immunohistochemistry and 173 other special stains - mainly PAS, Prussian-blue, picrosyrius, Masson's trichrome) were scanned. No smears or cytology samples were scanned. The cases were submitted to the pathologists as usual. PathA/B/D only received cases, specific to their field. Pathologist C/E/F/G received non-field specific cases too, including skin, thyroid and the GI-tract samples. PathH (junior) received cases from this latter pool of samples.

**Table 1:** Enrolled cases according to the localisation (n)

bone marrow	10
breast	25
colorectum	32
kidney	30
liver	25
lung	22
lymphnode	18
skin	58
soft tissue	22
thyroid gland	16
upper GI-tract	48

### Hardware and software tools used

Slides were scanned using 3Dhistech (3DH) Scan 1.11 equipped with a Hitachi 3-chip camera, a Plan-Apochromat objective (20× magnification), 0.5× camera adapter magnification, resulting in 0.465 μm/pixel resolution. The slide format was *mrxs*, set for 80 JPEG Quality factor. Sample recognition during scanning was automated, the technicans only loaded the slides. For data management we used 3DH DataBase (DB) software. (Additional file 1: Figure S1) No direct connection were built between the DB and the LIS. For the office PCs used in the study the usual set up of was the following: 1.6 GHz Intel - Dual CPU, 1 GB-RAM, 19" monitor with a resolution setting for 120 dpi, and 32 bit color mode.

### The evaluation process

1. Participating pathologists reached their assigned cases via the DB.

2. Initially, only those slides that were available for the first assessment were uploaded.

3. DS of all subsequent cuts, stains and IHC-reactions as in routine practice were uploaded on demand. When a stain had not been requested for the OM diagnoses, re-cut, staining and slide scanning were available.

4. Pathologists rendered microscopic descriptions and diagnosis to each case.

5. A Clinical Research Form was filled. (Table 2)

**Table 2:** Clinical research form

Scan quality	Explanation of the possible answers
*1-Unacceptable*	*critical deficiences (out of focus, missing scan) *
*2-Poor*	*major deficiences (large areas out of focus, missing parts)*
*3-Adequate*	*region of interests are proper, minor deficiences*
*4-Good*	*region of interests are focused, good color fidelity, minor deficiences *
*5-Excellent*	*whole material is focused, good color fidelity*
**The reason of dissatisfaction with scan quality**	**Polar questions**
*Important areas of the slide are out of focus*	*(y/n)*
*Incomplete scan*	*(y/n)*
*The color fidelity is poor*	*(y/n)*
*Other*	*(free text)*
**Diagnostic confidence**	**Explanation of the possible answers**
*1-Uncertain*	*consultation should be requested, no definite idea of diagnosis*
*2-Likely*	*consultation should be requested for confirmation*
*3-Confident *	*no consultation required*
**The reason of diagnostic uncertainty is due to**	**Polar questions**
*Case complexity*	*(y/n)*
*Poor image quality*	*(y/n)*

7. After all data were available a consensus session was held, consensus diagnosis were given for the cases and the missed cases were graded according to clinical significance of the error. (Table 3) The diagnostic errors was defined relevant when it had therapeutic or prognostic consequence and uncertainty was stated either because of case complexity or poor image quality recorded by the pathologist.

**Table 3:** Four types of incoherency

Type of diagnostic error	Description	number of cases (n)
Type I.	non relevant incoherence - uncertainty recorded	5
Type II.	non relevant incoherence - uncertainty not recorded	7
Type III.	relevant incoherence - uncertainty recorded	17
**Type IV**.	**relevant incoherence - uncertainty not recorded**	**7**

The clinical research form and the error grading system were designed with the purpose of simplicity and reproducibility, considering the literature [14]. The time taken to read the slides digitally versus optically was not measured.

## Results

### Technical results

**Figure 1:**
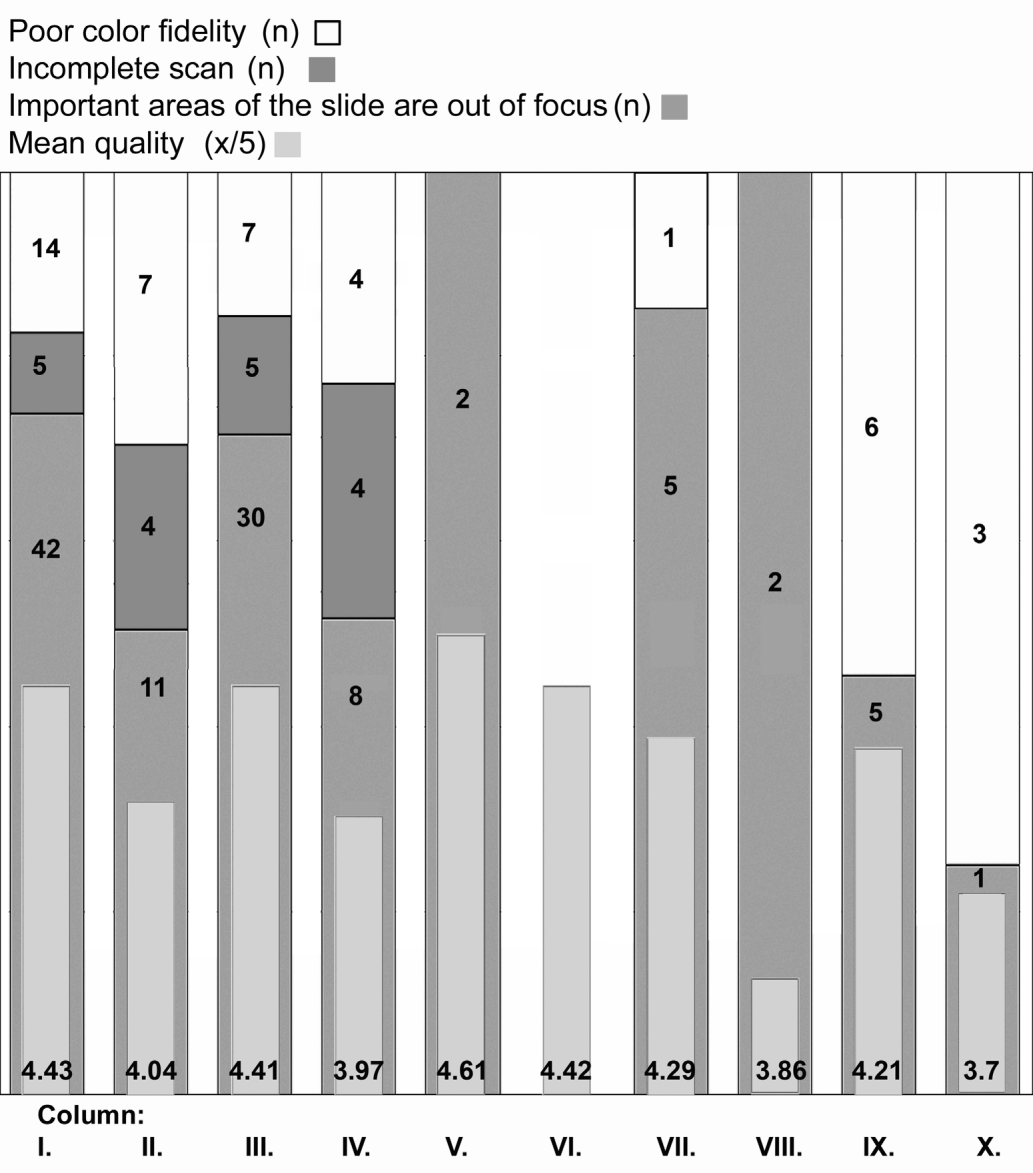
**Results of the slide quality query**. Column I.: all slides, C.II.: all slides of the incoherent cases, C.III.:all H&E slides, C.IV.: H&E slides of the incoherent cases, C.V.: all IHC slides, C.VI. IHC slides of the incoherent cases, C.VII.: all Giemsa slides, C.VIII.: Giemsa slides of the incoherent cases, C.IX.: all slides with other stains, C.X.: all slides with other stains of the incoherent cases.

The technical results of the scanning process are shown in Table 4. Out of the 1858 scanned slides 1621 were evaluated by the pathologists for digital diagnose, the remaining slides (special stains, IHC) were not asked. The average quality of the 1621 slides was 4.43/5. At 42 slides the reason of dissatisfaction was that "important areas of the slide were out of focus". 5 times the scan was considered incomplete and 14 times the color fidelity was rated poor. (Figure 1) Additional file 2: Figure S2, Additional file 3: Figure S3 and Additional file 4: Figure S4 show various examples for low quality images.

**Table 4:** Detailed parameters of the scanning properties of 1858 slides

number of slides ((n)	1858
scan time (min)	6824
FileSize (GB)	518
Area (cm2)	5193
area/scantime (cm2/min)	0,76

### Diagnostic results

DM or OM diagnosis and the consensus diagnosis were different in 63 (20.6%) cases (*discordant case*). In 36 (11.7%) cases (*incoherent case*) the OM and in 27 (8.82%) cases (*reassessed case*) the DM yielded the correct diagnoses. The mean diagnostic confidence was 2.7/3. Uncertainty due to case complexity was recorded in 48 (15.7%) cases, due to poor image quality in 15 (4.9%) cases. The results are detailed in 3 sections, according to our previous questions, focusing on the type of error, the origin of the samples and the pathologists, respectively.

### Types of error

**Figure 2:**
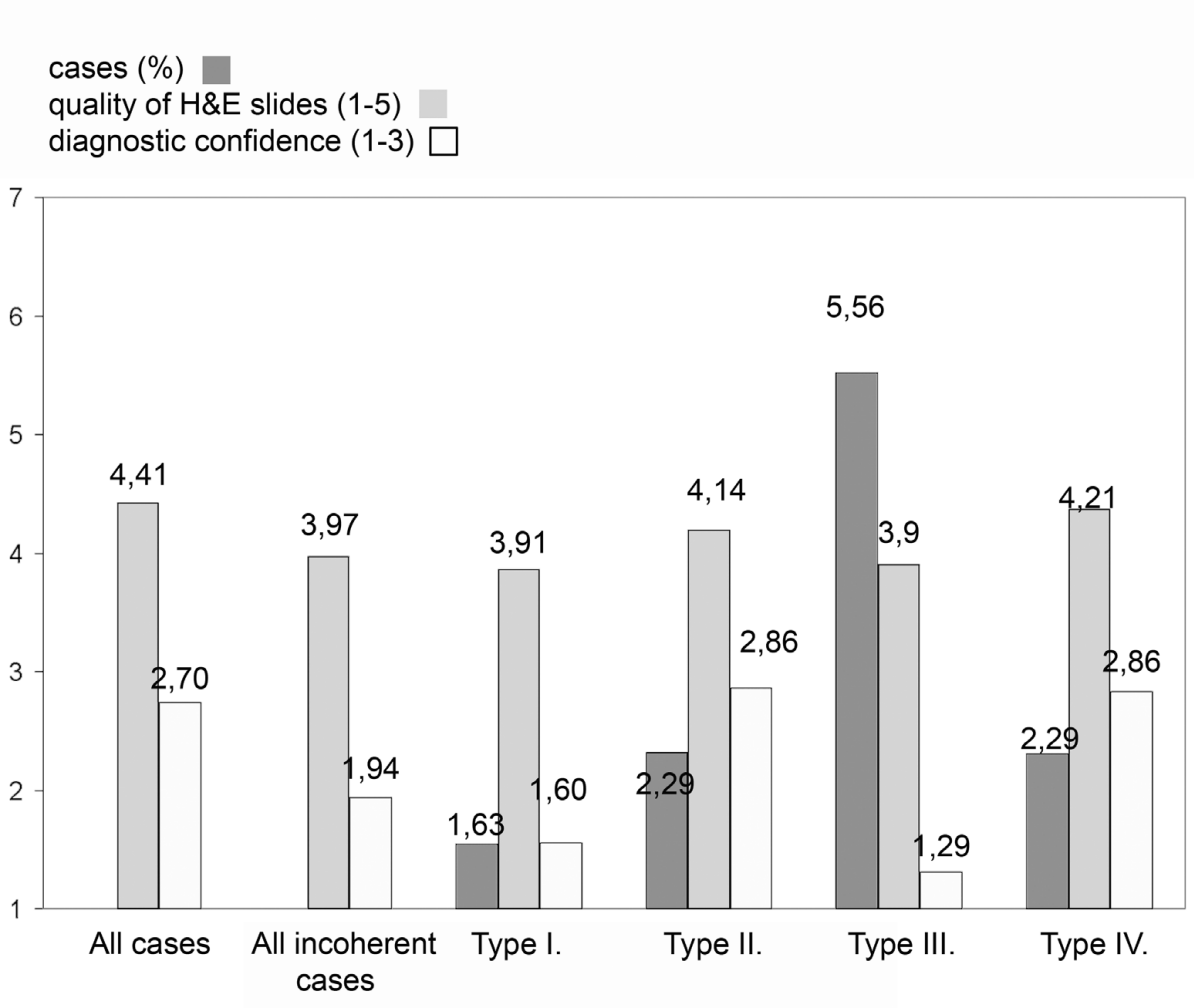
**Quality of H&E slides and diagnostic confidence according to the type of incoherency**.

The mean diagnostic confidence was 2.7/3 for all cases, whereas it was only 1.94/3 for the incoherent cases. Figure 2 summarizes the correlation of the diagnostic confidence and the quality of H&E slides according to type of error. (Additional file 5: Table S1 highlights the incoherent cases, digital and consensus diagnoses, diagnostic confidence and reasons of uncertainty.)

### Influence of sample origin

**Figure 3:**
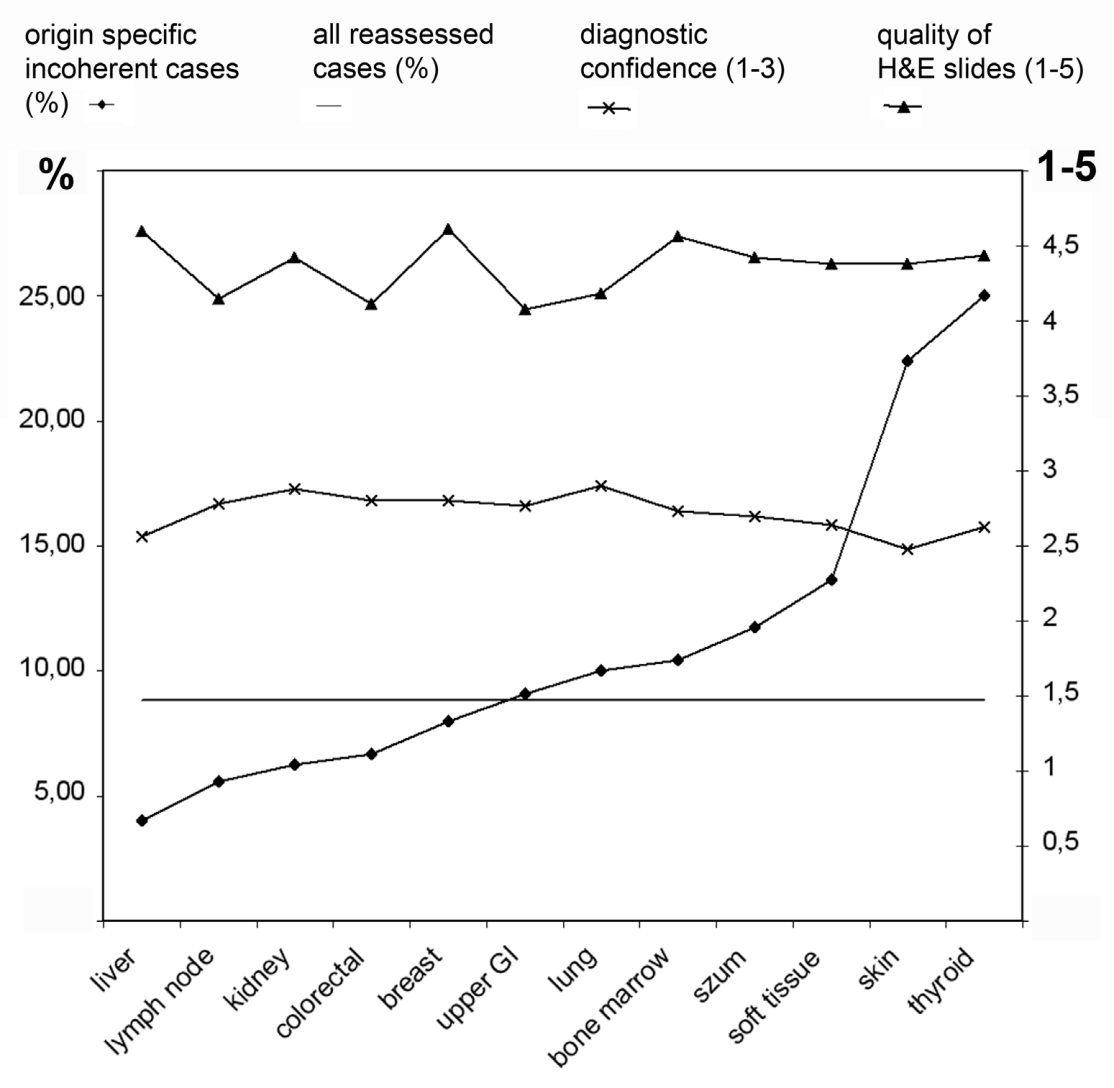
**Incoherency, quality of H&E slides and diagnostic confidence according to the origin of the sample**. In 8,82% of the cases the consensus diagnoses were coherent with the digital diagnoses and overwrote the original OM-based diagnoses (reassessed case - straight line)

The best coherency was found with liver samples where only one Type-III. error occured. Relatively poor results were given with bone marrow, thyroid and soft-tissue cases. Most uncertainty due to poor image quality were recorded in thyroid and upper GI samples. Figure 3 summarizes the correlation of the ratio of origin specific incoherent cases with the ratio of all reassessed cases, diagnostic confidence and quality of H&E slides according to the origin of samples. (Additional file 5: Table S2 shows the detailed results concerning sample origin.)

### Pathologists' competence

**Figure 4:**
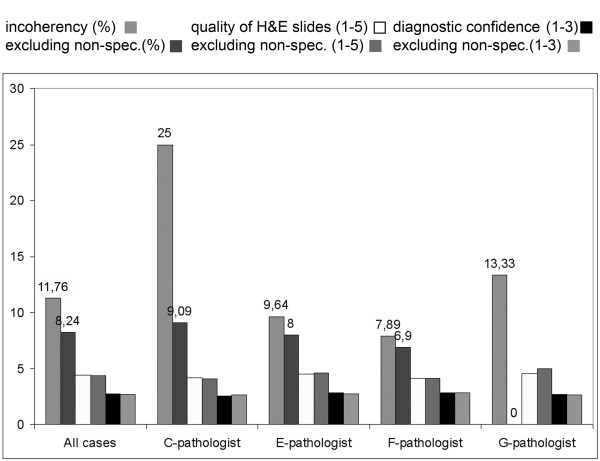
**Importance of pathologists' competence**. Excluding the non-field specific cases from each pathologists' record resulted in better coherency. No significant differences were found in the diagnostic confidence and how the pathologists rated the quality of the slides.

**Figure 5:**
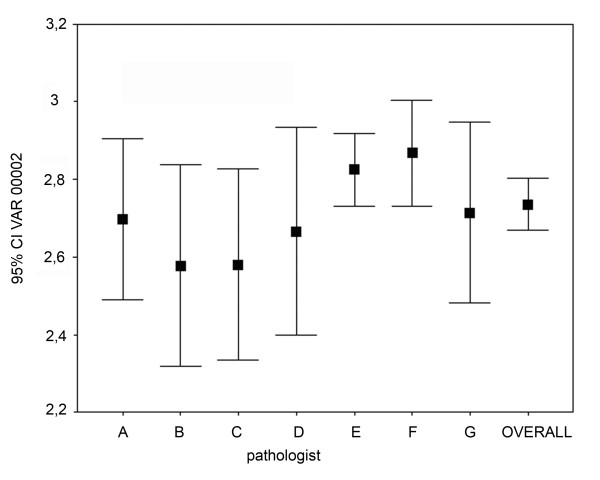
**Correlation between the experience (in years) and the diagnostic confidence**. overall = sum of all estimates. (Spearman rank R: R = -0.140, t(N-2) = -2.346, p = 0.019). Experience in years: PathA-20, PathB-25, PathC-22, PathD-24, PathE-13, PathF-15, PathG-28

We investigated the relation between the diagnostic results and the experience of pathologist and the impact of the pathologist's competence on various fields of histopathology. Excluding the non-field specific cases from each pathologists' record resulted in ~30% decrease in incoherency and in ~23% the Type-4 errors. Result of the most experienced pathologist (G-pathologist - 28 years) excluding the non-field specific cases was faultless. Second in this rank with 96% coherency was the second most experienced pathologist (B-pathologist - 25 years). (Figure 4) Interestingly we found a significant negative correlation between the experience (in years) and the diagnostic confidence (Spearman rank R: R = -0.140, t(N-2) = -2.346, p = 0.019). (Figure 5) Not surprisingly PathH (junior pathologist) gave the worst results, with 2.39 diagnostic confidence and inchorency rate over 16%. (Additional file 5: Table S3 shows the detailed results concerning competence.)

### Pathologists' subjective views

The database system allowed pathologists to enter comments in a free-text-box. Two of the comments were remarkable. One interestingly deals with the poor image quality of the slides at low power magnification where typically larger structures are analyzed. Some lymph node samples of haematopathology were considered problematic because the histological patterns (ex. the expansion of the different zones that surround germinal centers) were sometimes blurred at software built images of magnifications, lower than 200× (original scan magnification). Another returning comment was about the user friendliness of the viewer software. It was revealed that the pathologists who used only the mouse to navigate on the DS considered the speed of work with DS slower than those who used the keyboard control options as well. No comments were recorded in relation to the lack of option for moving the slide in along the 3^rd ^dimension z-coordinate.

## Discussion

In this study the diagnostic reliability of a fully digital slide based system, comparing it with the routine conventional optical microscope procedure was evaluated. Our results are in line with previously published studies in the field, where authors reported 94-98% accuracy of digital diagnoses [15-18]. Besides analyzing the results according to the origin of the samples and the types of errors we measured the effects of the pathologists' interpretative skills and experience on the diagnostic results.

In 27/306 - 8.82% - the consensus diagnoses were coherent with the digital diagnoses and overwrote the original OM-based diagnoses (reassessed case). We defined a list of samples according to their origin where DM could be used securely, based on comparing the incoherency-ratio of the specific samples to the overall ratio of the reassessed cases. The incoherency-ratio was below 8.82% - therefore we state that DM could be used equivalently to OM in our Institute in this set of circumstance - in cases with samples from liver, lymph node, kidney, colon, and breast. In this series the results nicely correlate with the incidence of the type-IV errors and confirm our statement. Interestingly hematology cases of lymph nodes fell into this category. As the quality of IHC digital slides were evaluated very good, the explanation of this observation could be the explicit importance of the IHC-profiles in haematopathology. This series of samples are specific for our institute in this set of circumstances. A similar method could be useful for pathology departments in the future when introducing DM in the routine practice in order to maintain patient safety.

Our further investigations estimated the type of errors resulting misinterpretation. Despite the evolution of the scanner systems that resulted in significant acceleration in scanning speed and better digital image quality the DS itself plays an important role in the success of the diagnostic process [19]. The mean quality of DS was 4.43/5. Excluding the slides from the misdiagnosed cases this rate is higher, 4.48/5, taking into account only the slides of the incoherent cases the result is 4.04/5, for the Giemsa stained slides in misdiagnosed cases it is 3.86/5 which is the worst result in any of the measured slide set along with the 3.70/5 result of the special stains. (Figure 1) However we have not recorded any complaints because of missing the 3^rd ^dimension z-coordinate, solutions to provide the pathologist with fine focusing ability on the DS are on the way and may enhance the acceptance of DS [20].

As no errors was recorded due to misinterpretation of an IHC-DS, our results suggests that the scanner systems in our constellation are sufficient to produce suitable DS from IHC-samples, as others reported similar results and explain the success and spreading of DS-based automated IHC evaluation techniques [21-23]. The most common reason for rating slide quality poor, was that large areas of the slides are out of focus. As this default is detectable by the examiner it never resulted in type II or type IV error. Interestingly the special stains (such as the PAS, Grocott, Prussian-blue, orcein etc.) showed bad result and the reason for this was poor color fidelity in the majority of the cases.

According to our results one of the most important factor of the diagnostic accuracy using DS, is the pathologist's experience in a specific field. There is an increase of diagnostic accuracy signing out only field-specific cases by the pathologists. There was significant negative correlation between diagnostic confidence and individual pathologist's experience. These results indirectly suggests that the impact of the pathologist's age is a major factor for dislike and mistrust DS, as usually more experienced the pathologists the older they are.

## Conclusions

• Digital microscopes could replace optical microscopes in many fields of routine pathology practice.

• Each pathology departments have to test the chosen digital microscope system in advance for themselves in order to estimate the user (pathologist) satisfaction and diagnostic accuracy.

• Quality of the digital slides are important to achieve the best possible diagnostic accuracy but the failure of proper scanning and expected image quality will not endanger patient safety as such errors are detectable by the examiner.

• In our study we found the most important factor of diagnostic accuracy is the pathologist's experiance in a specific field.

## Competing interests

Béla Molnár is the owner of 3DHISTECH Ltd. Budapest, László Fónyad and Tibor Krenács has commercial relationship with 3DHISTECH Ltd. as members of the advisory board for software development, they are no shareholders of the company. Other authors declare that they have no competing interests.

## Authors' contributions

LF, TK and BM conceived the study design and participated in case selection. PN, AZ, JC, ZS, JP, JS and CD contributed to the analysis of the digitally revised cases and participated in the consensus sessions. LF and TK performed data analysis. All authors were involved in the preparation of the manuscript and gave final approval of the submitted and published versions. All authors read and approved the final manuscript.

## Supplementary Material

Additional file 1 **Figure S1. User interface of the Mirax DataBase software**. Field 1. shows the Available Projects, where the cases submitted to the specific pathologist is listed, allowing the pathologist to organize the cases by setting up four different statuses, such as: New, Examined, Diagnosed or Reopened. First every DS were presented in a separate List of New Slides. (Field 2.) Later, any newly requested and uploaded slide (recuts, special stains, IHC) appeared here for limiting the chance of not using any single slide before signing out. In the Project Database and Preview (Field 3.) the Scanned Slides and their metadata (slide properties, attachments, direct links to slide annotations) can be seen. Field 4., as a slide-box, shows the thumbnail view of the slides of the active case. In. Field 5. clinical data and the gross description of the specimen is given in addition with results of the prehistological examinations such as cytology. Results of special diagnostic procedures, such as flow cytometry, molecular diagnostics, electronmicroscopy etc., were also edited here. Click here for file

Additional file 2 **Figure S2. Incomplete scan**. (Case 6., H&E, digital diagnose: fibrosis mammae; consensus diagnose: sine morbo) Click here for file

Additional file 3 **Figure S3. Scan is out of focus**. (Case 45., H&E, digital diagnose: chronic bronchitis, adenocarcinoma?; consensus diagnose: chronic bronchitis) Click here for file

Additional file 4 **Figure S4. Scan is out of focus, poor color fidelity**. (Case 226., Giemsa, digital diagnose: moderate chronic, active, aspecific gastritis; consensus diagnose: severe chronic, active, HP-associated gastritis) Click here for file

Additional file 5 **Table S1. Incoherent cases**. According to the *type of incoherency *the table highlights the origin of the samples, pathologist, and original and consensus diagnosis as well as data about diagnostic confidence from the *Clinical Research Form*. **Table S2. Influance of sample origin**. The table shows the collected data according to sample origin, highlighting organs where the organ specific incoherency ratio is below 8.82% (ratio of all reassesed cases). **Table S3. Pathologist competence**. The table shows the collected data on how the diagnostic confidence, reasons of uncertainty and ratio of the diagnostic errors changed after excluding non-field specific cases from each pathologists' record. Click here for file
