# Supplementary figures and images for: Validation of diagnostic accuracy using digital slides in routine histopathology
Source: Diagn Pathol. 2012 Mar 31;7:35. doi: 10.1186/1746-1596-7-35 (PMC3337227; doi:10.1186/1746-1596-7-35)

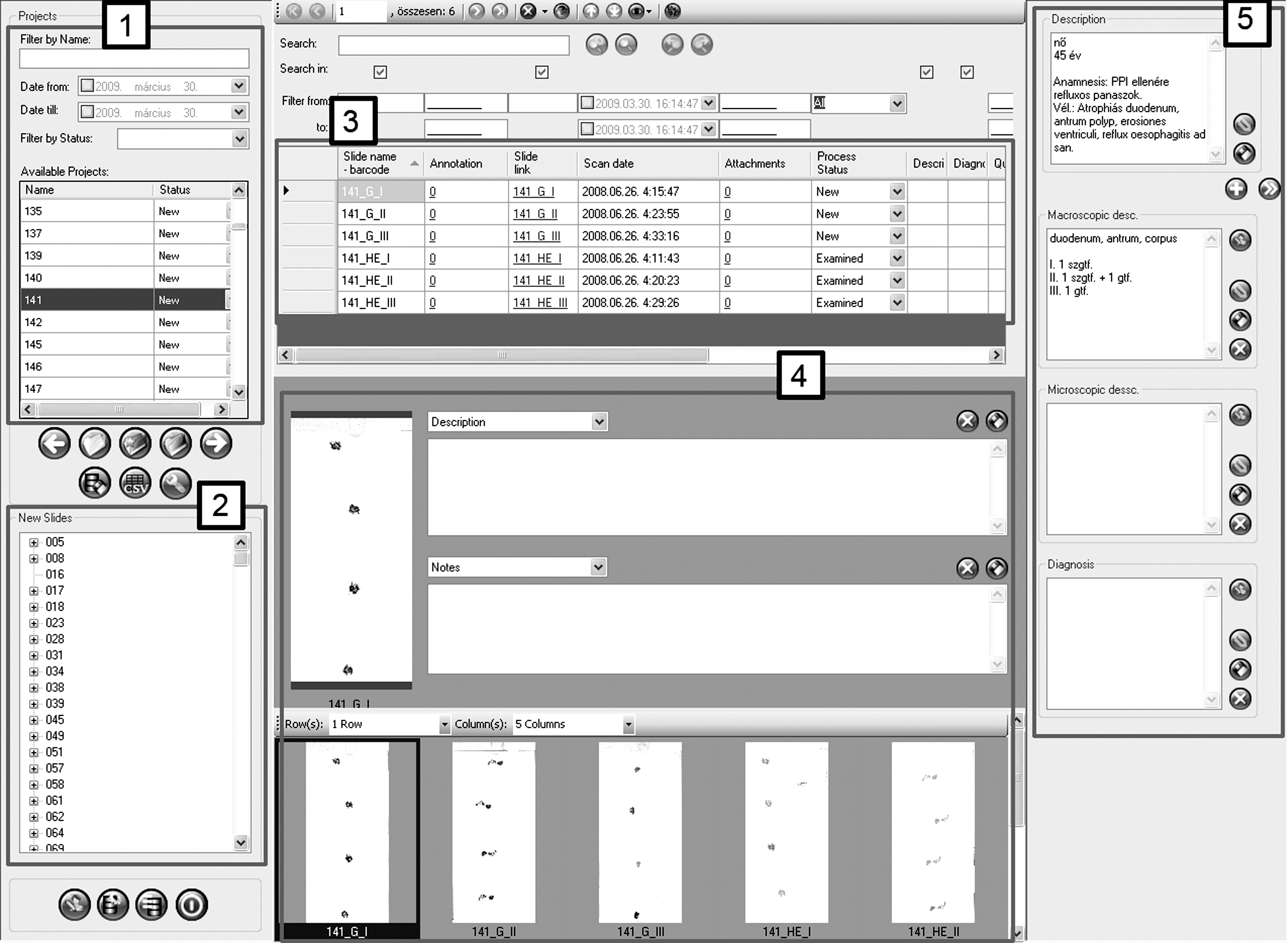

Supplement: Additional file 1 — Figure S1. User interface of the Mirax DataBase software. Field 1. shows the Available Projects, where the cases submitted to the specific pathologist is listed, allowing the pathologist to organize the cases by setting up four different statuses, such as: New, Examined, Diagnosed or Reopened. First every DS were presented in a separate List of New Slides. (Field 2.) Later, any newly requested and uploaded slide (recuts, special stains, IHC) appeared here for limiting the chance of not using any single slide before signing out. In the Project Database and Preview (Field 3.) the Scanned Slides and their metadata (slide properties, attachments, direct links to slide annotations) can be seen. Field 4., as a slide-box, shows the thumbnail view of the slides of the active case. In. Field 5. clinical data and the gross description of the specimen is given in addition with results of the prehistological examinations such as cytology. Results of special diagnostic procedures, such as flow cytometry, molecular diagnostics, electronmicroscopy etc., were also edited here. [file 1746-1596-7-35-S1.TIFF]

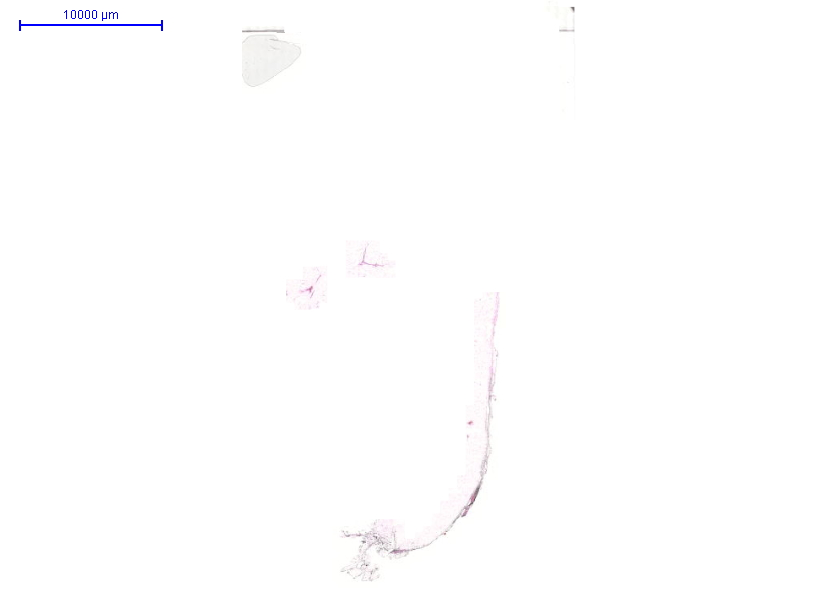

Supplement: Additional file 2 — Figure S2. Incomplete scan. (Case 6., H&E, digital diagnose: fibrosis mammae; consensus diagnose: sine morbo) [file 1746-1596-7-35-S2.TIFF]

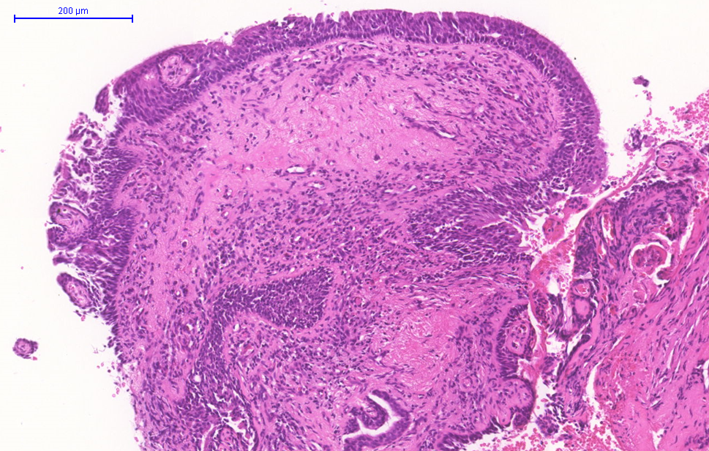

Supplement: Additional file 3 — Figure S3. Scan is out of focus. (Case 45., H&E, digital diagnose: chronic bronchitis, adenocarcinoma?; consensus diagnose: chronic bronchitis) [file 1746-1596-7-35-S3.TIFF]

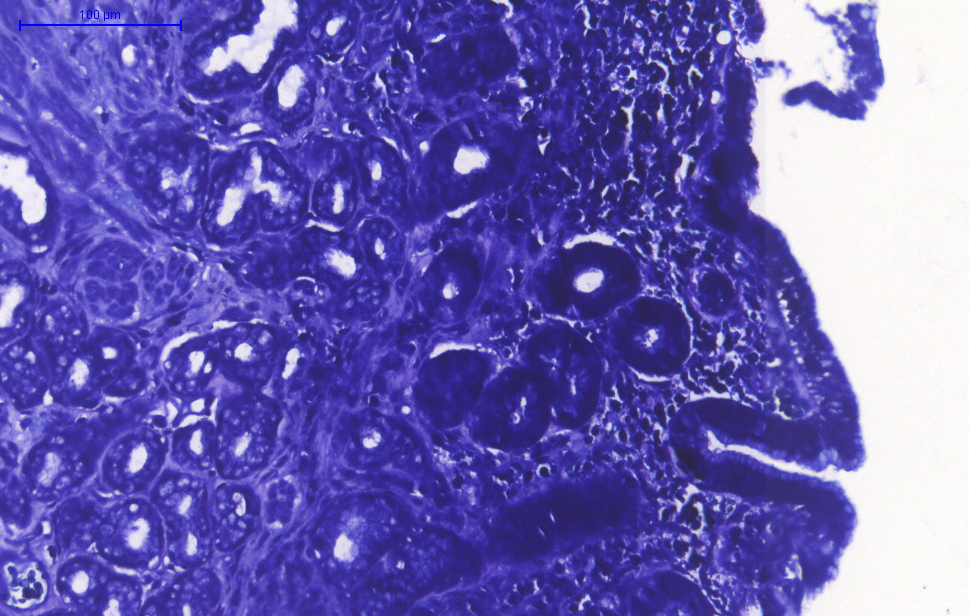

Supplement: Additional file 4 — Figure S4. Scan is out of focus, poor color fidelity. (Case 226., Giemsa, digital diagnose: moderate chronic, active, aspecific gastritis; consensus diagnose: severe chronic, active, HP-associated gastritis) [file 1746-1596-7-35-S4.TIFF]
